# Observations on Bronchotomy for the Removal of Foreign Bodies

**Published:** 1822-11

**Authors:** Michael Ward

**Affiliations:** Member of the Royal College of Surgeons, and Late Surgeon to the Manchester Infirmary, &c.


ART. VI.—*Observations on Bronchotomy for the Removal of Foreign Bodies.* By MICHAEL WARD, M.D. Member of the Royal College of Surgeons, and late Surgeon to the Manchester Infirmary, &c.

IN the twelfth volume of the Transactions of the Medico-Chirurgical Society, lately published, we meet with "A Case of Bronchotomy, successfully performed for the Removal of a Pebble from the Trachea, by WM. J. HUNT, M.D. Communicated by H. EARLE, Esq.; with Observations by H. EARLE, Esq."

In the course of Mr. Earle's observations, he says, "As a valuable additional fact in support of the practice which was so ably pursued, I felt anxious that the case should not be consigned to oblivion, *more particularly as I am not aware of any analogous case published in this country*."*

The remark I have just quoted induces me to request a page in your Journal, partly with a view to put Mr. Earle in possession of a valuable and important chirurgical fact, with which it appears he is unacquainted,† but principally in justice to my friend, Mr. Whitley, of Halton in Cheshire, who merits great praise, not only for the able manner in which he performed the

---

* *Medico-Chirurgical Transactions*, Vol. xii. Part i. p. 32.
† *London Med. and Phys. Journal*, No. 2 of Vol. xxxix. p. 100.

operation, but for the courage and perseverance with which he overcame the prejudices he had to encounter previous to its performance.

As an additional testimony, if any were necessary, of the complete and permanent success of the operation, I may add that I have been lately much gratified by a sight of the cicatrix in the neck of Mr. Whitley's patient, and the damson-stone which was removed from the trachea. The boy is now eleven years old, and in perfect health.

*Manchester; August 20th,* 1822.

